# Need for guidelines on prevention of abuse in the health-care sector

**DOI:** 10.2471/BLT.21.287555

**Published:** 2022-05-02

**Authors:** Tamim Alsuliman, Angie Mouki, Walaa Abdul Rahman

**Affiliations:** a Saint Antoine Hospital AP-HP, Sorbonne Université, Paris, France.; b Faculty of Pharmacy, Maykop State Technological University, Zhukovskogo Street 12, Maykop, Adygeya, 385000, Russian Federation.; c Directorate of Education, Tishreen University, Lattakia, Syria.

Bullying is a multifaceted form of mistreatment and is a growing issue in the health-care sector.^1^^,^^2^ This type of mistreatment is characterized by the repeated exposure of one person to physical and/or emotional aggression including teasing, name calling, mockery, threats, harassment, taunting, hazing, social exclusion or rumours.^3^ In 2002, the World Health Organization in collaboration with the International Labour Office, the International Council of Nurses and Public Services International published guidelines for addressing workplace violence in the health-care sector.^1^ The guidelines provide several recommendations for governments and employers to prevent bullying in the workplace. While many governments have not implemented these recommendations, others stepped forward and set frameworks for the prevention of bullying in the workplace. For example, the government of Western Australia implemented a zero-tolerance policy towards bullying under the employment policy framework in 2016, which was updated in 2019.^4^ The employers’ organization for the National Health Service in England has adopted guidance to address the issue of bullying from an organizational approach, providing tools and offering support to employees who experienced bullying.^5^ In addition, workers are encouraged to follow these guidelines and to report incidents of bullying. 

The guidelines also suggest that professional bodies and organizations create mechanisms and approaches to reduce workplace violence and eliminate associated risks. Many unions across the world, such as the Canadian Nurses Association, have taken actions and released supporting positions calling for violence-free workplaces.^6^

Nevertheless, the global response to the guidelines’ recommendations seems to have been heterogeneous. We argue that most concerned entities seem hesitant to apply the suggested measures. This hesitation could be because addressing workplace violence has not been prioritized, or because of ignorance of the importance of a violence-free work environment or lack of financial means.

Studies have found a disparity of results regarding the effectiveness of workplace violence treatment and prevention. A recent study reported a decrease in workplace violence when an integrative violence prevention approach combining education and training was implemented.^2^ On the other hand, others concluded that while studies show a slightly positive impact, evidence is sparse.^7^

The guidelines recommend that several principles be considered as organizations build an approach to tackle workplace violence: the approach should be integrated, participative, gender- and culturally sensitive, and non-discriminatory.^1^ Moreover, it should target the roots of the issue, and be implemented in a systematic manner. 

However, a few issues have arisen concerning these guidelines, including their structure and application. Most importantly, they are not up to date with the current challenges facing the health-care sector. For example, the guidelines do not address the emergence of new methods of workplace violence and bullying, namely their cyber form (that is, through social media, messaging platforms, gaming platforms or mobile phones). 

The guidelines developed few, if any, intervention protocols for cyberbullying. Moreover, the guidelines have not been updated with a clear definition of cyberbullying in the workplace, and they do not offer any recommendations for addressing workplace cyberbullying. 

The coronavirus disease 2019 (COVID-19) pandemic has increased cases of workplace violence, specifically of health-care worker bullying.^8^ The increasing occurrence of this phenomenon led to higher levels of anxiety in health-care workers, which in turn negatively influenced their mental health. The high level of anxiety in health-care workers is suggested to impact patient safety and lead to more medical errors.^9^ Additionally, bullying of health-care workers negatively affects attendance and increases employee turnover rates. These issues can have detrimental effects on an overwhelmed health-care system. Health-care workers experience both in-person and cyber forms of workplace violence, including incidents with patients and co-workers. However, only half of health-care workers who have been a victim of bullying have reported it.^8^

Doctors in training experience among the highest levels of bullying within the health-care system, probably because they are in a transitional phase between being students and professionals, and are the most accessible link between the general population and health-care institutions. A meta-analysis published in 2020 found that, in 52 studies, the overall pooled workplace violence is prevalent in more than half of residents where the most common forms of workplace violence were verbal, physical and sexual.^10^ Resident doctors are also more exposed to COVID-19 patients, because they are on the frontlines of emergency departments and medical services. They might therefore be more exposed to those who are critical of the health-care system’s response to the pandemic – more so during the first months of the pandemic. 

Additionally, during the pandemic, medical students and health-care workers of Asian descent experienced increased real-life and cyberbullying due to the presumed geographical origin of the virus. Increased use of social media played a major role in the wave of xenophobia against people of Asian descent, causing higher rates of cyberbullying.^11^

Moreover, higher rates of bullying have been recorded in certain demographics within the health-care sector in general. For instance, female health-care workers have reported higher rates of bullying than their male counterparts.^12^

The mounting evidence of bullying and workplace violence against health-care workers suggests that previously functional prevention and treatment methods are not as efficient anymore, probably because these measures have not been implemented fully or systematically. New bullying tactics need new preventive strategies.

Therefore, we call for an updated version of guidelines for addressing workplace violence in the health-care sector. In this new version, we suggest creating flexible and adaptable frameworks where fragile health-care systems can create their own approaches to tackle this issue. Developing a new version of the guidelines is important and timely, as fragile health-care systems have fewer resources, and face worsening working conditions and increasing workplace violence.

To reinforce the implementation of the guidelines and ensure implementation quality, we suggest creating a new national and/or international health-care institution scoring system based on workplace violence prevention and treatment. Such a scoring system would probably have a positive effect on both patients and health-care workers. Studies have found that a safe and healthy work–hospital environment promotes patient convalescence and decreases the number of medical mistakes through ensuring that health-care workers are in a good mental and physical state.^9^

A well-structured framework to monitor, prevent and treat cyberbullying is needed. We propose that those health-care workers affected by bullying be consulted during the process of guideline development, as their input can prove useful in developing applicable and experience-based approaches and guidelines for tackling the issue of workplace violence in the health-care sector.

We also highlight the need to improve the quality of the work environment and of prevention of workplace violence approaches by modernizing our communication systems through the better application of easy-to-use social media platforms. Coordination between governments, employers, workers, professional bodies and organizations is essential. However, such coordination must be complemented by a transparent and continuous evaluation of actions – and their outcomes – taken by the directors and governing bodies of health-care institutions. Finally, adapting and enforcing new guidelines to protect vulnerable categories and demographics of health-care workers is also necessary. 

## References

[1] Framework guidelines for addressing workplace violence in the health sector. Geneva: International Labour Office; 2002. Available from: https://www.ilo.org/wcmsp5/groups/public/---ed_dialogue/---sector/documents/normativeinstrument/wcms_160908.pdf [cited 2021 Nov 1].

[2] Somani R, Muntaner C, Hillan E, Velonis AJ, Smith P. A systematic review: effectiveness of interventions to de-escalate workplace violence against nurses in healthcare settings. Saf Health Work. 2021 Sep;12(3):289–95. 10.1016/j.shaw.2021.04.004 34527388 PMC8430427

[3] Srabstein JC, Leventhal BL. Prevention of bullying-related morbidity and mortality: a call for public health policies. Bull World Health Organ. 2010 Jun;88(6):403. 10.2471/BLT.10.077123 20539848 PMC2878162

[4] Prevention of Workplace Bullying Policy. Perth: Government of Western Australia, Department of Health; 2019. Available from: https://ww2.health.wa.gov.au/About-us/Policy-frameworks/Employment/Mandatory-requirements/Human-Resource-Management/WA-health-system-policies/Prevention-of-Workplace-Bullying-Policy [cited 2021 Nov 1].

[5] Bullying in healthcare [internet]. London: NHS Employers; 2019. Available from: https://www.nhsemployers.org/articles/bullying-healthcare‬‬ [cited 2021 Nov 1].

[6] Workplace violence and bullying - Joint position statement. Ottawa: The Canadian Nurses Association (CNA) and the Canadian Federation of Nurses Unions; 2008. Available from: https://nursesunions.ca/wp-content/uploads/2019/10/Workplace-Violence-and-Bullying_joint-position-statement.pdf [cited 2021 Nov 1].

[7] Wirth T, Peters C, Nienhaus A, Schablon A. Interventions for workplace violence prevention in emergency departments: a systematic review. Int J Environ Res Public Health. 2021 Aug 10;18(16):8459. 10.3390/ijerph18168459 34444208 PMC8392011

[8] Devnani M. COVID-19 and laws for workplace violence in healthcare. BMJ. 2021 Nov 15;375(2776):n2776. 10.1136/bmj.n2776 34782360

[9] Purdy N, Spence Laschinger HK, Finegan J, Kerr M, Olivera F. Effects of work environments on nurse and patient outcomes. J Nurs Manag. 2010 Nov;18(8):901–13. 10.1111/j.1365-2834.2010.01172.x 21073564

[10] Bahji A, Altomare J. Prevalence of intimidation, harassment, and discrimination among resident physicians: a systematic review and meta-analysis. Can Med Educ J. 2020 Mar 16;11(1):e97–123. 10.36834/cmej.57019 32215147 PMC7082478

[11] Li Y, Galea S. Racism and the COVID-19 epidemic: recommendations for health care workers. Am J Public Health. 2020;110(7):956–7. 10.2105/AJPH.2020.305698

[12] Ariza-Montes A, Muniz NM, Montero-Simó MJ, Araque-Padilla RA. Workplace bullying among healthcare workers. Int J Environ Res Public Health. 2013 Jul 24;10(8):3121–39. 10.3390/ijerph10083121 23887621 PMC3774428

